# Main Pathogenic Mechanisms and Recent Advances in COPD Peripheral Skeletal Muscle Wasting

**DOI:** 10.3390/ijms24076454

**Published:** 2023-03-29

**Authors:** Pauline Henrot, Isabelle Dupin, Pierre Schilfarth, Pauline Esteves, Léo Blervaque, Maéva Zysman, Fares Gouzi, Maurice Hayot, Pascal Pomiès, Patrick Berger

**Affiliations:** 1 Centre de Recherche Cardio-Thoracique de Bordeaux, Univ. Bordeaux, U1045, F-33604 Pessac, France; isabelle.dupin@u-bordeaux.fr (I.D.); schilfarth.pierre@gmail.com (P.S.); pauline.esteves@u-bordeaux.fr (P.E.); maeva.zysman@u-bordeaux.fr (M.Z.); patrick.berger@u-bordeaux.fr (P.B.); 2 INSERM, Centre de Recherche Cardio-Thoracique de Bordeaux, U1045, CIC 1401, F-33604 Pessac, France; 3 CHU de Bordeaux, Service d’Exploration Fonctionnelle Respiratoire, CIC 1401, Service de Pneumologie, F-33604 Pessac, France; 4 PhyMedExp, INSERM-CNRS-Montpellier University, F-34090 Montpellier, France; leo.blervaque@gmail.com (L.B.); pascal.pomies@inserm.fr (P.P.); 5 PhyMedExp, INSERM-CNRS-Montpellier University, CHRU Montpellier, F-34090 Montpellier, France; f-gouzi@chu-montpellier.fr (F.G.); m-hayot@chu-montpellier.fr (M.H.)

**Keywords:** cachexia, interleukin, metabolism, myocyte

## Abstract

Chronic obstructive pulmonary disease (COPD) is a worldwide prevalent respiratory disease mainly caused by tobacco smoke exposure. COPD is now considered as a systemic disease with several comorbidities. Among them, skeletal muscle dysfunction affects around 20% of COPD patients and is associated with higher morbidity and mortality. Although the histological alterations are well characterized, including myofiber atrophy, a decreased proportion of slow-twitch myofibers, and a decreased capillarization and oxidative phosphorylation capacity, the molecular basis for muscle atrophy is complex and remains partly unknown. Major difficulties lie in patient heterogeneity, accessing patients’ samples, and complex multifactorial process including extrinsic mechanisms, such as tobacco smoke or disuse, and intrinsic mechanisms, such as oxidative stress, hypoxia, or systemic inflammation. Muscle wasting is also a highly dynamic process whose investigation is hampered by the differential protein regulation according to the stage of atrophy. In this review, we report and discuss recent data regarding the molecular alterations in COPD leading to impaired muscle mass, including inflammation, hypoxia and hypercapnia, mitochondrial dysfunction, diverse metabolic changes such as oxidative and nitrosative stress and genetic and epigenetic modifications, all leading to an impaired anabolic/catabolic balance in the myocyte. We recapitulate data concerning skeletal muscle dysfunction obtained in the different rodent models of COPD. Finally, we propose several pathways that should be investigated in COPD skeletal muscle dysfunction in the future.

## 1. Introduction

Chronic obstructive pulmonary disease (COPD) is a global burden and is currently the third cause of death worldwide [1]. It is a chronic respiratory disease primarily linked to inhaled noxious particles exposure, mainly tobacco smoke, but also biomass fuel in developing countries [2], although recent research increasingly considers the role of different risk factors in very early life (i.e., in utero and during early childhood) in the development of COPD [3]. Lung features of COPD include both airway obstruction, currently used to diagnose the disease and the disruption of alveolar walls called emphysema [4]. However, COPD should not be limited to its respiratory features and is now considered as a systemic inflammatory disease implicating a wild range of cytokines and chemokines [5] and including several comorbidities.

Among them, skeletal muscle wasting is a prevalent and severe condition, affecting up to 35% of the patients depending on the considered population (ambulatory or nursing-home-based), and responsible for substantial morbidity and mortality [6,7]. Skeletal muscle dysfunction in COPD patients can be referred to in several ways. On a semantic point of view, different terms can be found in the literature, which should be distinguished from each other. Sarcopenia is a specific form of muscle loss diagnosed through a loss of muscle force and mass and is associated to several conditions other than COPD, primarily aging [8]. Muscle atrophy or wasting is a general term defining a reduction in the size of the muscle fibers, caused by an increased catabolism, whereas muscle dysfunction rather refers to a loss of force. Finally, cachexia is a specific metabolic syndrome associated with an ongoing underlying disease. These different concepts can however be interrelated, especially in the case of COPD, where patients are often of advanced age and suffering from many comorbidities, such as cardiovascular diseases, metabolic syndrome, or cancer, which are well-known causes of cachexia.

The origin of skeletal muscle wasting in COPD is still questioned, in particular whether it only results from external factors (such as tobacco smoke and disuse) or also from specific pathophysiological mechanisms bound to COPD itself. In particular, physical inactivity is a major confounding factor, challenging to rule out in comparative studies including healthy subjects and COPD patients. As a result, no specific and efficient pharmaceutic therapy is available to date, and pulmonary rehabilitation remains the only validated treatment [9]. Indeed, skeletal muscle wasting is challenging to study due to several limitations. First, there is limited access to patients’ samples, as muscular biopsy is not part of routine care. Some muscles are not easily accessible outside the context of lung cancer surgery, such as the diaphragm, and the coexisting condition introduces a notable bias as cancer is a well-known contributor of muscle wasting. In particular, inverse structural changes are usually reported in respiratory muscles, such as an increase in type I fiber proportion, capillary, and mitochondrial density; hence, this review focuses on alterations reported in peripheral muscles, as excellent reviews already address the differences between respiratory and peripheral muscle (see [10] for example). Secondly, the origin of muscle wasting is most likely to be multifactorial and is challenging to assess when limited samples are available for a heterogeneous group of sarcopenic patients, ranging from obese to malnourished. Moreover, local factors further complicate the issue: peripheral muscles are actually a heterogeneous group comprising locomotor muscles (mainly lower limb muscles) and upper limb muscles, both submitted to different force constraints. To date, studies performed in COPD patients have mainly focused on the quadriceps and more precisely vastus lateralis muscles. Lastly, muscle wasting is a dynamic process where the same molecules can be up- or downregulated according to the regenerative stage. Assessing the stage of disease evolution can be useful to harmonize patients’ samples; however, no clinical criteria are validated regarding the stage of muscle function impairment.

Several clues can be provided by experimental animal models of COPD, which allow access to every muscle as well as time-course studies.

In this review, we recapitulate the current knowledge regarding the molecular alterations leading to a decreased muscle mass and function, gathered around an impaired muscle mass regulation (unbalanced protein synthesis and degradation) and decreased oxidative capacity (mitochondrial dysfunction). The identified factors for skeletal muscle wasting are multiple and range from extrinsic factors such as disuse, malnutrition, or corticosteroids use, to intrinsic factors, such as inflammation and hypoxia. We report recent pathophysiological advances, with a special focus on preclinical models.

## 2. Histopathological Alterations of COPD Skeletal Muscle Tissue

**Figure 1:**
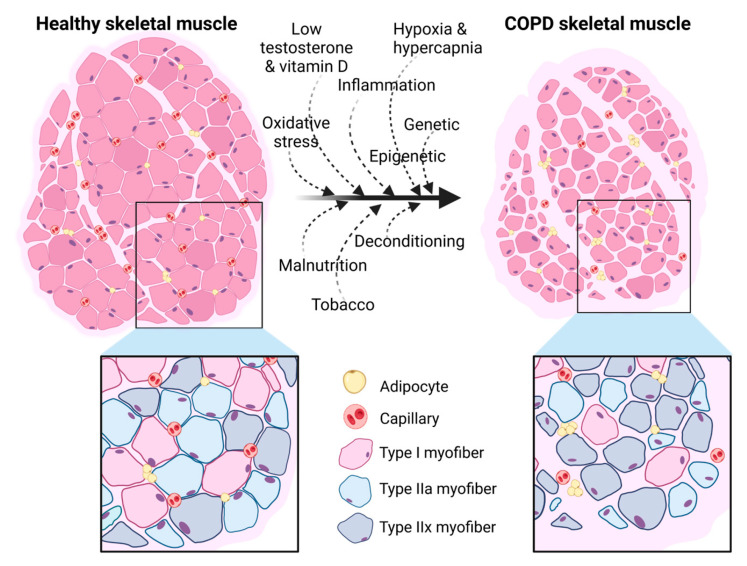
Schematic representation of the main histopathological alterations in COPD skeletal muscle. Alterations of skeletal muscle tissue in COPD result from both extrinsic factors (malnutrition, tobacco exposure, deconditioning) and intrinsic factors (hormonal imbalance, oxidative stress, inflammation, hypoxia and hypercapnia, and genetic and epigenetic modifications). Created with BioRender.com (accessed on 1 February 2023).

Structural alterations of peripheral skeletal muscle are well characterized and include a global muscle atrophy (with both loss in number and size of muscle fibers), macroscopically reflected by a decrease in thigh diameter [11] and microscopically reflected by a decreased fibers’ cross-sectional area (CSA) [12] (Figure 1). A specific metabolic alteration in COPD muscle (as opposed to aging muscle for example) is the shift in fiber type, from fatigue-resistant oxidative (type I and type IIa) to force-predominant glycolytic muscle fibers (type IIx and, specifically for rodents, type IIb) [12] (Figure 1). The decreased fibers’ CSA has been reported to be predominant on type IIx fibers [12]. Other known histological alterations include a decreased capillarity, with both a decrease in capillaries density as well as in the capillary-to-fiber ratio [13] (Figure 1). Capillarization has notably been found impaired specifically in type I muscle fibers, which has a deep impact on the muscle’s oxidative capacity [14,15]. Adipose tissue infiltration has also been reported in COPD muscle, in a similar way to senescent muscle in the aging population, particularly in the obese sarcopenic phenotype where adipose inflammation leads to the redistribution of fat to the intra-abdominal area and skeletal muscle tissue [16,17] (Figure 1).

## 3. Inflammation

The historical perspective is that inflammation is one of the primary drivers of skeletal muscle wasting in COPD, through the catabolic effects of systemic inflammation. Indeed, it has been hypothesized that COPD muscle atrophy would be a consequence of the “spill over” of inflammatory molecules from the lungs into the systemic circulation [18]. However, inflammation is not the sole driver as exercise can affect factors regulating muscle hypertrophy and regeneration without affecting the levels of systemic or local muscle inflammation [19]. Moreover, it is now increasingly recognized that inflammation in muscle homeostasis bears both beneficial and detrimental effects. Chronic, low-grade inflammation induces muscle catabolism via pleiotropic mechanisms mediated by the inflammatory secretome [20], whereas acute inflammation (exercise-mediated or in acute muscle injury models) activates anabolic pathways leading to muscle regeneration. Several soluble factors secreted by muscle cells, named myokines, take part in muscle mass homeostasis. This section focuses on myokines and recent data regarding their role in COPD skeletal muscle; for a general review on each cytokine, see [21] or [22]. Studies globally consistently show that COPD is characterized by the elevation of inflammatory factors such as tumor necrosis factor α (TNF-α), interleukin-6 (IL-6), and interleukin-8 (IL-8).

### 3.1. Tumor Necrosis Factor α (TNF-α)

Classically, circulating TNF-α is reported to be higher in COPD patients than controls and negatively correlated to lean mass [23,24], as well as to muscle strength, including upper muscles [25]. However, this correlation seems to be consistent only in cachectic patients [26]. Moreover, with regards to muscular TNF-α, significantly elevated, decreased, and unaltered levels have been reported in the quadriceps of COPD patients [27,28,29], or even undetected at the protein level [30,31], although the effects of TNF-α could also be mediated by the elevated circulating levels. Mechanistically, TNF-α plays a dual role in muscle mass regulation, possibly via differential signaling according to receptor binding (TNF-R1 or TNF-R2) [32,33]. On the catabolic counterpart, TNF-α promotes the inflammatory response by binding on TNF-R at the macrophage surface and activating the nuclear factor-kappa B (NF-кB) pathway via the inhibitory-kappa B kinase alpha (IKKα) activation, thus supporting the M1-biased (inflammatory) phenotype. TNF-α secreted by myeloid cells (CD68+ macrophages) inhibits myotubes fusion in vivo [34], supporting its catabolic role. In vitro, it can be induced in C2C12 myotubes by serum amyloid A, which is increased during COPD exacerbations [35]. Possibly in a paracrine/autocrine fashion, its secretion activates glycolytic metabolism in C2C12 myotubes in a NF-кB-dependent manner, as well as enhancing the secretion of HIF1α, a key transcription factor induced in hypoxic or inflammatory conditions [36]. In the same study, HIF1α and its downstream target vascular endothelial growth factor (VEGF) were found elevated in quadriceps biopsies of COPD patients and correlated to the level of muscular TNF-α. Finally, a recent study has found that the downregulation of histone deacetylase 2 (HDAC2) by RNA interference in cultured primary myotubes increased TNF-α production and cell apoptosis [37]. HDAC2 belongs to a family of enzymes which classically downregulate inflammatory genes’ expression. The authors also evidenced a decrease of HDAC2 level in the quadriceps of COPD patients, associated with an increase in the NF-кB pathway.

However, some in vivo models seem to counteract in vitro data. Indeed, a mice model of TNF-α-signaling knock out (TNF-R2 KO) exhibited a more severe phenotype of skeletal muscle wasting (including reduced fibers’ CSA and an increase of type IIx myofibers) than wild-type littermates [32] after cigarette smoke (CS) exposure. Other data support the anabolic effects of TNF-α, which acts as a mitogen for satellite cells [38]. In myocytes, the binding of TNF-α to its receptor TNF-R activates the p38-mitogen-activated protein kinase (MAPK) pathway, leading to a silencing of target genes such as Pax7 and Notch1 (via an epigenetic control), promoting satellite cell differentiation into myotubes [39], in contrast to that previously reported [40]. The latter study also showed an increase in mitochondrial content in IKKα-expressing myotubes. Other models tend to corroborate in vitro data, such as a mouse model exposed to CS and exhibiting increased circulating TNF-α levels, which were correlated to detrimental outcomes such as muscle mass, decreased capillarization, and increased catabolism [41]. Moreover, an overexpression of TNF-α in the lung leads to an increased muscle fatigue, decreased muscle mass and mitochondrial content, and increased catabolism (mostly in male mice) [42]. These data might suggest that part of the deleterious effects of inflammation are secondary. Of note, therapies against TNF-α have not proven to be effective for various outcomes such as dyspnea or a number of exacerbations [43], suggesting that this cytokine is not the primary or at least the sole driver of COPD inflammation.

### 3.2. Interleukin-6 (IL-6)

Circulating IL-6 is also classically elevated in COPD patients compared to healthy subjects, both during and outside exacerbations [44] and associated with reduced quadriceps strength [45]. Like TNF-α, IL-6 is considered as a “double-edged sword” with proinflammatory (catabolic) and anti-inflammatory (anabolic) effects (see the review in [46]) according to local immune cells and the cytokines’ microenvironment. Overall, the pilot study of Tsujinaka and colleagues showed that transgenic mice chronically overexpressing IL-6 exhibit a marked muscle atrophy [47]; however, the knockout of IL-6 was not sufficient to prevent sarcopenia in a sepsis mouse model [48]. In muscle from COPD patients, IL-6 has been reported as unchanged at the transcriptional level (at both stable and exacerbation states) compared to that of healthy controls [27,28].

In vitro, IL-6 bears anabolic effects by regulating satellite cells’ function and enhancing glucose metabolism; furthermore, a loss of IL-6 signaling in myoblasts results in a reduced proliferation and migration [49,50].

### 3.3. Interleukin-8 (IL-8)

In muscle from COPD patients, IL-8 has been reported as unchanged at the transcriptional level (at both stable and exacerbation states) compared to that of healthy controls [28]. Surprisingly, despite an important role for IL-8 in COPD pathophysiology and in particular lung inflammation, few data are available regarding its expression in the skeletal muscles of COPD patients.

### 3.4. Interleukin-18 (IL-18)

One study has found an elevation of IL-18, another proinflammatory cytokine, in the plasma and the quadriceps of COPD patients (at the mRNA level) compared to healthy controls [51]. Immunohistochemistry showed that the increase was predominant in type II fibers. Of note, muscle mRNA levels of TNF-α and IL-6 were unchanged in that study. Moreover, IL-18 mRNA levels were not significantly different in the quadriceps of COPD patients compared to that of healthy smokers. Of note, contrary to IL-6, IL-18 was not altered by exercise [51]. It was hypothesized that this increase could participate in skeletal muscle wasting by increasing local apoptosis.

### 3.5. Interleukin-15 (IL-15)

In vitro, IL-15 has an anabolic effect by inducing myosin chain synthesis in myotubes [52], notably in response to TNF-α stimulation [53]. This is particularly interesting given that IL-15 was increased and correlated with TNF-α in a rat model of COPD (induced by CS exposure as well as LPS instillations), in the serum and in both peripheral and respiratory skeletal muscles [54]. In the same study, this was paralleled by an increase in ubiquitin–proteasome markers (as expected), which were positively correlated to IL-15 levels. Furthermore, IL-15 possibly promotes the effector function of memory CD8+ T cells [55]. However, no data have been reported in human COPD muscle samples to the best of our knowledge.

### 3.6. Interferon-γ (IFN-γ)

IFN-γ has been reported as unchanged at the protein level in COPD quadriceps compared to controls [27]. However, a recent transcriptional analysis shows a globally downregulated interferon response in COPD quadriceps [56]. In vitro, IFN-γ does not activate catabolic pathways on C2C12 myoblasts [57] but induces the expression of proangiogenic factors such as angiopoietin-2 in primary human myoblasts [58]. Taken together, these data may indicate that IFN-γ downregulation could be part of the pathological mechanisms leading to skeletal muscle wasting, although other observational as well as functional data lack at this stage.

### 3.7. Interleukin-10 (IL-10)

IL-10 exerts a consistent anti-inflammatory, pro-regenerative role (as demonstrated by acute injury models). It bears potent anabolic effects by inhibiting the atrophy signaling induced by TNF-α in myotubes [59]. As a cytokine implicated in muscle regeneration in acute injury models (see below), its potential protective role in muscular dystrophy is not surprising [60]. However, to date, it has not been studied in skeletal muscles of COPD patients.

### 3.8. Other Myokines

Other myokines can also play a role in the control of muscle mass, most of which having not been thoroughly investigated in COPD, such as IL-4 (promoting myoblast fusion in vitro) [61], IL-7, which secretion by skeletal muscle gradually decreases with age [20], or irisin, known to decrease oxidant-induced apoptosis in diabetes mellitus, and recently reported to be decreased in COPD serum [62]. Two studies outside the COPD context also point towards a prominent role of the Toll-like receptor (TLR)-4 pathway in muscle wasting, via the activation of the p38-MAPK atrophy pathway [63,64]. However, to date, the expression of IL-17, a cytokine activating TLR4 signaling, has not been investigated in COPD muscle.

## 4. Hypoxia and Hypercapnia

Chronic hypoxia in healthy subjects (implicated in altitude expeditions) induces a loss of muscle mass and decreased fibers’ CSA and mitochondrial density, without affecting the capillary network [65]. Hypoxia seems to play a prominent role in the skeletal muscle wasting of COPD patients, as suggested by a network-analysis transcriptomic studies evidencing upregulation of four histone deacetylase in the muscle of COPD patients (HDAC 9 and 4, SIRT 2 and 3) compared to that of healthy subjects and correlated with oxygen availability [66].

In experimental models, hypoxia clearly induces muscle wasting, as evidenced by several reports showing muscle atrophy even after only few days of hypoxia exposure [67,68]. At the structural level, hypoxia might favor a fiber-type switch in the muscle of COPD patients [69]. Several hypotheses have been formulated regarding the molecular basis. In the study of Favier and colleagues, hypoxia was followed by a downregulation of the anabolic pathway’s mechanistic target of rapamycin (mTOR) [67] (see Section 9.2). A recent study showed an inhibition of the AKT/GSK3-β/P70S6K protein synthesis pathway associated with an increased autophagy-related LC3BII/LC3BI ratio in C2C12 myotubes cultured in hypoxic conditions [70]. Some studies also found a direct link between hypoxia and the catabolic ubiquitin–proteasome pathway [71]. Along the same line, hypoxia was found to increase myostatin (a negative regulator of muscle mass) expression in a rat model [72]. Conversely, hypercapnia led to AMPK/FoxO3a/MuRF1-dependent muscle fiber size reduction in an experimental model [73].

The transcription factor hypoxia-inducible-factor-1α (HIF-1α) is a major effector of hypoxic pathways. Surprisingly, very few data are available regarding its expression in the muscle of COPD patients. To our knowledge, only one study investigated HIF-1α expression and found an increased mRNA level in the quadriceps of COPD patients, related to increased TNF-α signaling [36]. Studies unrelated to COPD explored the molecular basis for HIF-1α in skeletal muscle wasting. HIF1-α expression increases in C2C12 myotubes submitted to hypoxia [70]. In C2C12 myoblasts, HIF-1α inhibits the canonical Wnt signaling known to promote muscle regeneration [74]. Moreover, HIF-1α activates the MAP kinase catabolic signaling pathway’s extracellular signal-regulated kinase (ERK1/2) (cf. infra) [75]. All these mechanisms need to be confirmed in the muscle of COPD patients. Some effects of hypoxia are HIF-1α-independent [76], and interestingly, low levels of HIF-1α in skeletal muscle resulted in a more altered phenotype in a mouse model, with an impaired exercise response [77], indicating either that hypoxia-mediated deleterious effects could be independent of HIF-1 α, or that HIF-1α elevation activated a compensatory mechanism.

Hypoxia also seems to alter myonuclear turnover by affecting satellite cells’ (muscle stem cells) activity and myogenesis [76]. In an experimental model of hindlimb ischemia, hypoxia induced a reduced protein expression of the skeletal-muscle differentiation markers (myogenic differentiation 1 (MyoD) and myogenin) [78]. These observations were in line with that already shown in vitro [79]. Hypoxia also alters the terminal maturation of myotubes by decreasing myosin heavy chain’s expression [71]. CD34^+^ muscle stem cells are specifically affected by hypoxia: Pagé and colleagues found a worse outcome for CD34^−/−^ mice exposed to hypoxia compared to wild-type littermates, with a lower number of fibro-adipogenic progenitors in CD34^−/−^ mice and a greater muscle atrophy as well as a worse functional impairment of the extensor digitorum longus muscle [68]. These observations were associated with a lower number of fibro-adipogenic progenitors in CD34^−/−^ mice. Collectively, these data indicate that muscle stem cells are affected by hypoxia, albeit the precise mechanisms remain to be elucidated.

Hypoxia might also be the main reason for impaired COPD muscle response to exercise training. A study showed that exercise training improved functional capacity in both normoxemic and hypoxemic COPD patients, however the structural improvements (increased mitochondrial density, capillary-to-fiber ratio, and fibers’ CSA) occurred only in the normoxemic ones [80]. This might be due to increased oxidative stress, specifically in the hypoxemic population [30]. Intricate mechanisms with malnutrition could also compete, since exposing mice to hypoxia leads to a loss of appetite [81]. However, matching mice for food uptake still showed a deleterious effect of hypoxia on protein turnover via the downregulation of mTOR signaling (cf. infra) [82]. Finally, a recent study pointed out a possible role of intermittent hypoxia (such as the nocturnal hypoxemia seen in obstructive sleep apnea disorder, frequent among COPD patients [83]) in skeletal muscle wasting, driven by mitochondrial dysfunction and a lower activity [84].

Overall, hypoxia is an important contributor of muscle wasting, although direct mechanistic evidence in COPD muscle is lacking.

## 5. Mitochondrial Dysfunction

An early clinical translation of muscle wasting is increased fatigability, exercise intolerance, and a diminished endurance. Thus, it is not surprising that mitochondria appear to be at the center of sarcopenia pathophysiology [85,86]. Mitochondrial alterations include a decreased mitochondrial density (biogenesis), impaired mitochondrial oxidative capacities (activity) and mitochondrial autophagy (mitophagy), increased apoptosis, and finally, an increased mitochondrial reactive oxygen species (ROS) production (oxidative stress).

### 5.1. Reduced Mitochondrial Density/Dynamics

The main determinant of the reduced mitochondrial activity observed in COPD patients is the decreased mitochondrial content associated with a loss of type I fiber and oxidative phenotype. Thus, data consistently show a decreased mitochondrial density in COPD muscle tissue, usually reflected by a decrease in citrate synthase expression or activity, as a mitochondrial mass marker [87]. As another surrogate marker, mitochondrial DNA over genomic DNA ratio (mtDNA/gDNA) is also lower in COPD muscle compared to healthy controls [88]. However, data are less consistent at the individual protein level. The expressions of electron transport chain (ETC) complex proteins I, II, III, and V have, for example, been reported as unchanged in patients with mild COPD (GOLD II) but decreased in patients with severe or very severe COPD (GOLD III-IV) [89].

Transcript levels of regulators of mitochondrial biogenesis have been described both as upregulated and downregulated in COPD compared to controls. Remels and colleagues observed low levels of peroxisome proliferator-activated receptor-gamma coactivator 1 alpha (PGC1-α, the master regulator of mitochondrial biogenesis) and its downstream effector, mitochondrial transcription factor A (TFAM), in COPD quadriceps; these differences were further enhanced in cachectic COPD patients [90]. However, Konokhova and colleagues found an upregulation of mitochondrial biogenesis markers transcripts, including PGC1α and TFAM, but also peroxisome proliferator-activated receptors (PPARs), but not at the protein levels. Other authors suggested an impaired translation in COPD quadriceps [87]. Interestingly, PGC1-β overexpression was responsible for muscular atrophy due to an enhanced apoptosis and autophagy in a transgenic mouse model [91]. In vitro data tend to reproduce the former results, as mitochondrial biogenesis markers PGC1α (and to a lesser extent TFAM and COX1) and MFN2 have been shown to be reduced in cultured myotubes from COPD patients compared to healthy subjects upon electrical stimulation [92].

Konokhova and colleagues also performed a single fiber analysis of cytochrome c oxidase/succinate dehydrogenase coloration (COX/SDH) and did not find any evidence of mitochondrial biogenesis impairment in COPD COX-/SDH+ fibers. Indeed, the mtDNA copy number and TFAM protein content of COX−/SDH+ fibers were similar between COPD patients and healthy subjects. Of note, more frequent mtDNA deletions and lower mtDNA copies were observed in COPD patients’ quadriceps compared to controls. Moreover, contrary to normal aging, there was no more mtDNA copies in the COX+/SDH+ fibers compared to COX-/SDH+ fibers in COPD patients.

A recent study explored the possibility of the reduced mitochondrial density being due to deconditioning by matching COPD patients to control subjects for objectively measured physical activity [93]. The results showed that the dysfunctional mitochondrial phenotype observed in COPD patients could not be attributed to deconditioning alone, as evidenced by a decreased citrate synthase activity, altered mitochondrial respiration, and increased oxidative stress in COPD patients compared to control subjects with the same level of physical activity.

Relatively few data are available concerning the mechanisms of a reduced mitochondrial network. Only one study investigated dynamin-1-like protein DNML1 (also known as DRP1, a GTPase regulating mitochondrial fission) expression in COPD quadriceps and found a decreased protein level [88].

### 5.2. Reduced Mitochondrial Activity

In a cohort of 79 COPD patients, Willis-Owen and colleagues recently performed a nonbiased transcriptomic analysis and showed that COPD was accompanied by coordinated patterns of transcription of genes involved in mitochondria function in the quadriceps [94]. In particular, the transcriptome revealed an important lower expression of IDH2. IDH2 is the enzyme responsible for the oxidative decarboxylation of isocitrate to α-ketoglutarate, and its lower activity is largely described in oncology, where it leads to a reduced metabolic activity [95].

Mitochondrial activity can be evaluated either by the cytochrome oxidase (COX)—complex IV—activity, the terminal complex of the respiratory chain, or a respiratory state assessment. Results for the specific activity of COX normalized on mitochondrial density are conflicting. While some authors have found an increased COX activity in COPD patients with resting hypoxemia [96] and with exercise hypoxemia [97], others have not found any significant increase [98]. The reasons for such discrepancies may be due to different measurement methods, including the activity of the COX-IV determined spectrophotometrically or with immunohistochemical staining, and the respiratory capacities of complex IV using different substrates. Moreover, differences in the sampled subjects’ resting or exercise arterial blood oxygen content could be determinant for the measurement of oxygen respiration capacities. Interestingly, some COX genes such as COX11 or COX15 are upregulated after training in the quadriceps of COPD patients but not sedentary healthy controls [99].

Several reports, which are detailed below, point towards an altered mitochondrial respiration in ex vivo muscle experiments. The current knowledge considers that state 3 respiration (ADP-stimulated) is impaired in COPD patients; however, contradictory data are reported regarding complex I- (CI) or complex II-, succinate dehydrogenase (CII-SDH), driven respiration states [100].

The first study was performed in isolated mitochondria and showed a diminished state 3:CII respiration in low-body-mass-index (BMI) COPD patients compared to both normal BMI and healthy subjects [99]. Further studies were then performed in quadriceps’ permeabilized fibers. First, Picard and colleagues observed a decreased state 2 and state 3 respiration in COPD fibers compared to healthy controls. However, a normalization with respect to the citrate synthase activity abolished these differences [98]. Performing the same normalization, Naimi and colleagues found a similar state 3:CI+CII respiration, along with an increase in state 3:CI respiration, inferring a decrease in state 3:CII respiration. Finally, Gifford and colleagues observed a reduction in state 3:CI+CII respiration in COPD patients compared to healthy controls [101]. Of note, contrary to Naimi and colleagues, they concluded that this reduction was largely driven by diminished CI-driven respiration rather than CII-driven respiration. The same authors confirmed their observations in 2017 on a different cohort of COPD patients matched to healthy controls based on the same physical activity, in order to avoid disuse bias. They observed a diminished state 3 CI-driven respiration, and a trend to a lower state 3:CI+CII driven respiration. However, the normalization of respiration with respect to the citrate synthase activity again revealed no differences in any state 3 or state 2 respiration between COPD and healthy subjects, while a normalization by OXPHOS protein content revealed a significant increase in state 3:CII respiration in COPD patients. The authors globally concluded that CI/CII respiration ratio was altered in COPD muscle, although results were conflicting according to the normalization method. Overall, discrepancies between studies mainly come from two points: (i) the analysis performed either on isolated mitochondria or permeabilized fibers; (ii) the methods of normalization varying between authors. Further studies are warranted, in a standardized manner, in order to collect more data. Recently, Balnis and colleagues evidenced a reduced SDH (CII) activity underlying a decreased mitochondrial respiration in the extensor digitorum longis of a mouse model of emphysema (IL-13 transgenic mice) [102]. Importantly, transfecting the defective CII subunit C to mice muscles significantly increased both mitochondrial function (respiration) and oxidative myofibers’ proportion, indicating that mitochondrial function could regulate the muscle fiber phenotype in vivo. However, other observations tend to show that metabolic and contractile properties are controlled by distinct signaling pathways [103].

During exacerbations, mitochondrial respiration seems to be further decreased, as confirmed by a study investigating the transcriptomic profile of quadriceps biopsies between exacerbating COPD patients and stable COPD controls, and evidencing a decreased aspartate catabolism as well as COX6C activity [104]. Of note, mitochondrial respiration can be affected by external factors, such as glucocorticoid use [105], and the specific role of COPD in altered respiration remains to be elucidated.

### 5.3. Increased Mitophagy

Evidence of increased mitophagy, mainly attributed to CS exposure, has already been reported in the lung [106]. Data are more scarce in skeletal muscles, but the potential role of mitophagy in skeletal muscle wasting has been recently reviewed [107]. The first study was performed in a murine model of lung injury (induced by LPS instillation) and showed an enhanced transcription of mitophagy-related genes such as BNIP3L and PARKIN and a decrease of FUNDC1 [108]. The same authors simultaneously investigated COPD patients’ quadriceps biopsies and found decreased FUNDC1 and PINK1 at the protein level, whereas Parkin was increased. Moreover, no change was observed at the mRNA level [109]. These discrepancies reflect both the difficulty of obtaining experimental models as close as possible to the disease and the complex regulation of the mitophagy process. Finally, the same team performed a similar study in COPD patients according to their inflammatory status and found an increase in BNIP3 at the protein level in COPD patients with high circulating C-reactive protein (CRP) compared to other COPD patients, pointing out that mitophagy was associated with systemic inflammation [110].

### 5.4. Apoptosis

Regarding skeletal muscle cell apoptosis, contradictory data have been reported. Since the early reports of Agusti and colleagues and Gosker and colleagues, who observed, respectively, an increased and unchanged myonuclear fragmentation in muscle cells [111,112], only one study reported increased terminal deoxynucleotidyl transferase mediated dUTP nick-end labeling (TUNEL)-positive cells in quadriceps and diaphragm muscles [113]. However, no ultrastructural difference in apoptotic nuclei was noticed. Of note, there was no means to discriminate between myocytes and surrounding cells in these studies. Discrepancies might also come from the fact that Agusti and colleagues reported an increased apoptosis specifically in COPD patients with low BMI, whereas Gosker and colleagues investigated moderate-to-severe COPD patients without weight indication, and Barreiro and colleagues reported COPD patients with normal body composition. Noteworthy, these two studies failed to report an increased caspase-3 expression in COPD patients [112,113]. Surprisingly, few data are available to date in COPD despite the fact that caspase-induced apoptosis is a known contributor of muscle wasting [114].

Mitochondrial permeability transition pore (mPTP) is a key regulator of mitochondria during cell death [115]. Puente-Maestu and colleagues have reported an enhanced susceptibility to mPTP opening by common triggers (including Ca^2+^ and H_2_O_2_) in isolated mitochondria of COPD muscle cells [85]. However, and conversely, Picard and colleagues reported that there was more resistance to Ca^2+^-induced mPTP opening in permeabilized muscle fibers from COPD patients than in those from healthy control subjects, attributed to the increased proportion of type II fibers, which are intrinsically more resistant to Ca^2+^-induced mPTP opening [98]. These discrepancies are probably explained by the analysis performed on isolated mitochondria versus permeabilized fibers, which better reflect muscle function. However, they need to be confirmed.

## 6. Oxidative and Nitrosative Stress

Oxidative stress is increased both at the systemic and muscular levels in COPD [10,89,116]. In the muscle, ROS mainly originate from mitochondrial respiration and membrane (NAPDH oxidase). In the lung, by contrast, ROS are produced by alveolar macrophages and activated neutrophils [117]. Direct evidence includes increased nuclear and mitochondrial superoxide anions in the quadriceps of sarcopenic COPD patients compared to nonsarcopenic patients [118].

Since mitochondrial oxidative phosphorylation is incomplete, mitochondria are a major source of ROS production [119]. H_2_O_2_ production is significantly increased in the isolated mitochondria of COPD patients’ muscle compared to control subjects, in state 3:CI as well as state 3:CII respiration [85]. Indeed, the addition of rotenone, a specific inhibitor of complex I, validated the specificity of complex I’s site of production. The authors concluded that CIII was the main site of ROS production in COPD skeletal muscle and attributed this to the absence of matrix antioxidant in close vicinity (contrary to complex I). Picard and colleagues also showed in permeabilized fibers normalized per unit of citrate synthase activity that H_2_O_2_ release was significantly higher in COPD patients compared to control subjects during state 2 and state 3:CII [98]. The authors also confirmed the increased H_2_O_2_ production in COPD fibers in the presence of antimycin A. Of note, to date, no study has been able to settle the question whether the greater ROS production was solely the result of a fiber-type switch and sedentary lifestyle, as glycolytic fibers, where state 2 respiration is predominant, produce more ROS than oxidative fibers.

Alterations linked to excessive oxidative stress can be seen at several levels. The evidence for DNA damage is scarce but includes higher (8-OHdG) levels in COPD quadriceps compared to controls [87]. Alterations at the protein level include protein carbonylation, malondialdehyde (MDA)-protein adducts, lipid peroxidation, and nitration.

Multiple studies point towards an increased protein carbonylation in COPD quadriceps compared to healthy controls, including total protein content but also specific proteins such as glycolytic enzymes (creatine kinase), antioxidant enzymes (glutathione peroxidase), and myosin heavy chain [118,120,121,122]. MDA-protein adducts are also increased both at the muscular and systemic levels in COPD patients [123]. Intuitive functional implications arise from these observations, as oxidative stress is negatively correlated to contractile performance [124]. However, the absence of increased carbonylation in the quadriceps muscle of COPD patients compared to healthy controls has also been reported [125]. Increased lipid peroxidation has also been observed, possibly favored by reduced UCP3 levels [112]. For example, several studies have found an increased lipofuscin accumulation in COPD quadriceps [126] and higher levels of hydroxy-2-nonenal (4-HNE) in quadriceps tissue [87,127], as well as in cultured myotubes from COPD patients [128]. Ferroptosis, a newly discovered type of cell death resulting from iron-dependent lipid peroxide accumulation, has been reported to be increased both in vitro in CS-exposed C2C12 myotubes and in vivo in a mouse model of COPD [129]. Finally, nitrosative stress is also increased, as evidenced by higher tyrosine nitration levels in the quadriceps from COPD patients compared to those from healthy subjects [122], as well as inducible nitric oxide synthase and 3-nitrotyrosine levels [127].

Skeletal muscle tissue usually compensates the excessive oxidative stress (induced for example by exercise training) by increasing the levels of antioxidants [122,127], but in COPD, this protective mechanism seems to be defective, although contradictory data have been reported. For example, increased Mn-superoxide dismutase (SOD) and Cu-Zn-SOD protein levels have been demonstrated in the quadriceps [118,127], whereas unchanged SOD content and decreased SOD activity have been reported in another study [122], and unchanged MnSOD, Cu/ZnSOD, and catalase expressions in cultured COPD myoblasts and myotubes have been found [128]. A recent study evidenced a decreased in SOD2 quadriceps expression only in severe (GOLD III–IV) COPD patients [89]. However, data are more consistent for other protective mechanisms, which apparently fail to upregulate. Unchanged glutathione and catalase quadriceps levels have been reported [118,122]. Of note, we have shown an increase in glutathione peroxidase 1 in cultured COPD myoblasts [128]. Finally, the antioxidant protein carnosine, mainly present in type II myofibers, has been reported to be unchanged in the muscle of COPD patients compared to healthy controls; however, in COPD patients, carnosine was decreased in the quadriceps of GOLD III–IV patients compared to GOLD I–II, despite their increased proportion of type II myofibers [125].

Of note, some studies report no difference in oxidative stress markers between COPD patients and healthy smokers [130], implying that oxidative stress could mainly be attributed to CS extracts and not the specificity of systemic involvement of COPD.

No study has demonstrated a link between oxidative stress and inflammation in COPD muscle. However, several studies point towards a direct relationship between oxidative stress, atrophy, and the ubiquitin–proteasome pathway [131], including data obtained in experimental models [132]. It is also likely that oxidative stress enhances proteolysis via the calpain and caspase pathways [133], whereas this has not been directly demonstrated in COPD muscle.

Altogether, it seems that the predominant feature of COPD muscle is a transient antioxidant insufficiency, which brings hope in terms of therapeutic issues. For future therapeutic prospects, roflumilast, an inhibitor of phosphodiesterase-4 (PDE-4) which exerts antioxidant effects, has shown promising results in a proof-of-concept study on cultured myotubes from COPD patients [134]. Moreover, a combination of oral antioxidant supplementation has also shown interesting results on muscle strength with an excellent tolerance [135].

## 7. Other Metabolic Processes

Apart from oxidative and nitrosative stresses, other forms of metabolic stress have long been reported in COPD muscle. Alterations in amino acid (AA) profiles have been reviewed by Jagoe and Engelen [136] and include decreased glutamate and glutathione levels in COPD quadriceps [137]. These alterations seem to be further enhanced in emphysema patients with a decrease in almost all AA levels [138].

More recently, transcriptomic studies helped to uncover other metabolic differences between COPD patients and healthy subjects. Indeed, one study reported an increase in the COPD quadriceps of SLC22A3, a metabolic stress gene involved in AA transportation and histamine clearance [94]. Such increase had also been reported in COPD genome in genome-wide association studies (GWAS) [139]. Of note, the study of Adami and colleagues identified an SNP in the same gene, that was close to be significantly associated with muscle aerobic capacity response [140]. Willis-Owen and colleagues also reported an increase of FST, encoding follistatin, a propeptide inhibiting myostatin signaling and promoting myogenic differentiation. Thus, this upregulation might represent a compensatory mechanism. However, patients and controls were not matched for physical activity, unravelling a well-known bias due to deconditioning in COPD patients. Another recent study [56], using microarrays and gene ontology algorithms to detect network modules in COPD and healthy quadriceps, identified abnormalities in creatine metabolism and calcium homeostasis, which were significantly associated with exercise capacity. Specifically, the creatine metabolism pathway was significantly altered with downregulation of two genes related to creatine synthesis (GAMT, GATM) and two creatine kinase genes (CKB, CKMT2). These results point towards an impairment of the muscle energy production and are consistent with other studies showing low baseline creatine kinase and ATP concentrations [121]. The disruption of calcium homeostasis was represented by the downregulation of S100A1 gene, which could lead to abnormal sarcoplasmic reticulum Ca^2+^ content and fluxes, impacting muscle contractility [56]. Two other studies performed in CS-exposed mice evidenced impaired calcium signaling, notably a decreased sarcoplasmic reticulum calcium uptake [141,142]. To the best of our knowledge, no other study has investigated calcium signaling in COPD myocytes. However, a recent study reported elevated markers of sarcoplasmic reticulum stress (at the protein level) in COPD quadriceps [143]. Hyperammonemic stress is also known to be associated with COPD, and a recent study evidenced new pathways associated with hyperammonemia in skeletal muscle (in addition to the already-known mitochondrial dysfunction, oxidative stress, and senescence), such as antiapoptotic B-cell leukemia/lymphoma 2 family protein expression, increased calcium flux, and increased protein glycosylation [144]. Finally, a recent study evidenced elevated circulating kynurenine levels in COPD patients, as well as reduced muscle levels of kynurenine aminotransferases; this dysregulated kynurenine clearance was attributed to impaired PGC1α signaling [145].

On a therapeutic prospect, the metabolic modulator metformin has been shown to be able to protect against CS-induced myofibers’ type switch and telomere shortening in a mouse model of emphysema, mediated by the activation of 5′AMPK (AMP-activated protein kinase) and GDF-15 (growth differentiation factor 15) [146].

## 8. Genetic Predisposition and Epigenetic Modifications

Genetic predisposition, although not predominant, is a well-known risk factor for COPD development. Identified polymorphisms notably include genes involved in functions that can favor skeletal muscle wasting, such as oxidative capacity (glutathione-S-transferase, superoxide dismutase, etc.) or inflammation (TNF-α) [147]. However, few studies specifically investigate the relationship between genetic polymorphisms and skeletal muscle dysfunction in COPD. Some studies drew our attention.

Recently, a GWAS performed simultaneously on humans and mice allowed the identification of 23 loci associated with appendicular lean mass [148]. Among them, two genes were confirmed as in vitro modifiers of muscle mass, as their silencing with RNA interference increased the length of myotubes. These two genes, CPNE1 and STC2, encode proteins that have not yet been formally associated with COPD sarcopenia (i.e., Copine 1, a soluble calcium-dependent membrane-binding protein, and Stanniocalcin 2, a homodimeric glycoprotein hormone involved in the regulation of IGF1). Interestingly, STC2 is among the genes that are downregulated in skeletal muscle from mice following bimagrumab (activin type II receptor antagonist) treatment [149]. Future studies should aim to explore the expression of these proteins in skeletal muscle from COPD patients.

Furthermore, in 2010, a study conducted by Timmons and colleagues identified 11 single-nucleotide polymorphisms (SNPs) associated with the increase in maximal oxygen uptake (VO_2_max) after exercise training in the general population [150]. Later, Adami and colleagues investigated the association between the same SNPs and the muscle oxygen consumption recovery rate constant (k), assessed with near infrared spectroscopy, which reflects skeletal muscle oxidative capacity. Although no significant association was evidenced in COPD patients, three SNPs were associated with k specifically in moderate-to-severe COPD patients [140]. Among them, one SNPs was located in the DEPTOR gene, of particular interest as it is associated with the regulation of the contractile properties of glycolytic muscles [151].

Epigenetic modifications, whether methylation, acetylation, or muscle-specific micro-RNAs (myomiRNAs) play essential roles in muscle physiology, including embryogenesis and adaptation to environmental factors (for a comprehensive review, see [152]). Epigenetic modifications are believed to control muscle fiber type, and can be influenced by external factors such as nutrition [153]. Among them, miRNAs represent a growing body of interest in COPD, and they have been associated with several features of the disease in the lung [154]. However, few studies explore their expression in skeletal muscle, and data are until now contradictory.

Muscle-specific miRNAs (miR-1, miR-206, and miR-133) are associated with myotube formation and the innervation process. The pioneer study in the matter was conducted by Lewis and colleagues and showed a decreased expression of miR-1 in COPD quadriceps, as well as an increase in its target, histone deacetylase 4 (HDCA4) at the protein level [155]. The latter is thought to inhibit regulators of myosin heavy chain 1 expression, thus possibly accounting for the switch in fiber type. miR-1 is also associated with insulin-like growth factor-1 (IGF-1) regulation, providing a possible explanation for reduced protein synthesis (cf. infra). In that study, levels of miR-133 and miR-206 were unchanged. Later, miR-1, miR-206, and miR-133 were found significantly elevated in the plasma of patients with COPD, as well as miR-499, encoded within the slow myosin heavy chain (MHC) gene and thus promoting type I fibers [156]. Furthermore, levels of miR-499 were associated with nuclear NF-κB p50 in mild/moderate COPD, whereas in severe and very severe disease, miR-206 and miR-133 were associated with circulating cytokines, emphasizing the link with inflammation. However, in that study, although the authors performed muscular biopsies, they did not investigate the level of miRNA in the quadriceps, which would have allowed them to perform correlations with plasma levels. Then, Puig-Vilanova and colleagues found inverse results with a local decrease of miR-1 and miR-206 and an increase in HDAC4 in COPD quadriceps (including sarcopenic and nonsarcopenic patients) [157]. These discrepancies are possibly explained by the absence of matching for smoking and physical activity in the first study. Differences in timing of miRNA secretion can also be invoked.

Maternally imprinted genes are usually associated with limiting growth, whereas paternal genes bear the reverse effect [158]. An increase in the maternally imprinted miR-675 was found in the quadriceps of COPD patients with a low fat-free mass index (FFMI), and this was associated with an inhibition of myoblast proliferation in vitro [159]. Of note, no association between miR-675 and FFMI was found among healthy subjects. The authors then hypothesized that muscle wasting in chronic diseases might result from an evolutionary response in order to use muscle protein as a source of AA for the damaged organ or inflammatory system. Recently, miR542-3p/5p has been found elevated in COPD quadriceps. In vitro data showed its implication in mitochondrial stress and transforming growth factor beta (TGF-β) canonical signaling, and an in vivo experimental study confirmed its role in muscle wasting through a decrease of mitochondrial function [160].

Overall, further studies are warranted in order to harmonize findings for known muscle-specific miRNAs (miR-1, miR-206, and miR-133) as well as to identify other candidates. Their therapeutic potential should also be tested in this context. Finally, exosomal miRNAs also deserve further investigations, as one study (in abstract form) showed differentially expressed miRNAs in the serum and broncho-alveolar lavage fluid of COPD patients, which were associated with skeletal muscle wasting pathways such as mTOR [161].

## 9. Consequences on Muscle Mass Regulation

All mechanisms detailed above converge to deleterious consequences on muscle mass regulation, destabilizing the atrophy/hypertrophy balance. A consensual statement is that muscle protein turnover is impaired both through enhanced degradation and reduced synthesis. For a comprehensive review of potential triggers leading to impaired muscle mass regulation (including deconditioning and use of glucocorticoids), see [162].

### 9.1. Protein Degradation

#### 9.1.1. Implication of the 26S Ubiquitin–Proteasome System

The ubiquitin–proteasome system plays a major role in protein content turnover by degrading misfolded or defective proteins [163,164]. In COPD, increased proteolysis is mainly mediated by an overactivation of the ubiquitin (26S)–proteasome system, activated via the muscle-specific E3-ubiquitin ligases Atrogin-1/MAFbx and TRIM63/MuRF-1, also named atrogenes, which are increased in COPD quadriceps [118,165]. Of note, some studies have failed to report a significant difference between COPD patients and healthy subjects [166], possibly due to the timing of the assessment. The proteasome is regulated by the transcription factors FOXO1 and FOXO3; several upstream activators of FOXO signaling are increased in COPD muscle, such as the NF-кB pathway. During exacerbations, this pathway seems to be further enhanced in the quadriceps with the upregulation of MuRF-1 and Atrogin-1 expression [104]. Of note, this study was not conducted on the same patients (exacerbating versus stable COPD patients).

Nedd4, another muscle-specific E3-ubiquitin ligase acting through Notch1 signaling [167], has recently drawn attention because of its implication in denervation-induced muscle atrophy [168], here unrelated to Notch1 activation. Yet, its expression has not been thoroughly investigated in COPD, with only one report indicating an increased expression in COPD quadriceps [169].

#### 9.1.2. MAP-Kinase (MAPK) Pathways

MAPK pathways are classically considered as catabolic second messengers in myocytes, upon activation by different triggers such as inflammatory cytokines or ROS [170]. One study reported evidence of the activation of MAPK p38 and ERK1/2 in the quadriceps [171]. However, no upregulation was observed in two other studies [118,172]. Puig-Vilanova and colleagues reported an increase in p38-MAPK levels in COPD quadriceps but an unchanged ERK1/2 expression [123]. These discrepancies might come from the diversity in patients’ recruitment and warrant further investigations, although once again, the timing of the investigation is probably the most crucial point. Ultimately, p38-MAPK upregulation could directly lead to increased proteasome-related catabolism, as shown in mice [173].

#### 9.1.3. Myostatin

Myostatin is a ligand of the TGF-β pathway in skeletal muscle and a well-known negative regulator of muscle mass, as it inhibits satellite cells’ proliferation and differentiation [174]. Upon binding to its receptor, the activin receptor type II A/B, it activates catabolic pathways via the phosphorylation of downstream effectors Smad2/3 and simultaneously inhibits anabolic pathways [175]. Its expression is increased in the quadriceps of COPD patients [176], possibly related to hypoxia [72]. However, a recent clinical trial using a monoclonal antibody blocking its receptor has not shown potent results in treating skeletal muscle wasting for COPD patients [177], despite promising results previously obtained in cancer models [178].

#### 9.1.4. Autophagy

A major other proteolytic pathway is autophagy-induced lysosomal degradation, an essential process leading to the degradation of cytoplasmic portions of variable sizes in the lysosomes (see the review by [179]). It is already established that autophagy is increased in the lungs of COPD patients [180]. It also seems to play an essential role in skeletal muscle wasting, as muscle-autophagy-deficient mice (muscle-specific deletion of Atg7) exhibit sarcopenia features [181].

Autophagy can be induced by several physiological situations, such as fasting or physical exercise, and is a complex process requiring multiple simultaneous assays to assess. A classical way to evaluate autophagy is to assess the level of the autophagosomal membrane protein LC3 (microtubule-associated protein 1 light chain 3). Although conflicting results have been reported, the global conclusion is that autophagy is enhanced in COPD locomotor muscles. One study reported unchanged transcripts of LC3B and Beclin-1, another autophagosomal marker, in COPD quadriceps [169]. However, that study lacked the assessment of the LC3BII/LC3BI ratio (reflecting the active form) as well as that of other autophagy markers. By contrast, Hussain and colleagues reported an increase in quadricipital LC3BII, Beclin-1, and p62/SQSTM1 at the protein level [179]. After that, several reports pointed out an enhanced autophagy in COPD muscle, confirming the data observed in the lung [180]. A significant number of studies reported an increased LC3BII/LC3BI ratio and/or LC3B lipidation (another marker of activation) in COPD quadriceps [123,182,183]. An increased LC3BII/LC3BI ratio and p62/SQSTM1 protein levels were also reported in cultured myoblasts from COPD patients compared to healthy subjects [184].

More recently, Balnis and colleagues showed in satellite cells a defective autophagic flux associated with important proteins’ upregulation such as Beclin-1. In that work, autophagy seemed to be correlated to satellite cell dysfunction contributing to the pathogenesis of emphysema in a transgenic mouse model [185].

Furthermore, two studies reported increased ultrastructural autophagosome counts, respectively, in the quadriceps [123] and both in the quadriceps and tibialis anterior [182]. Guo and colleagues also reported an increase in the mRNA and protein levels of several autophagy-related genes, such as the genes involved in the autophagosome formation (BECN1, PI3KCIII, UVRAG, AMBRA1, GABARAPL1, ATG7), the selective targeting of mitochondria by autophagosomes (SQSTM1, BNIP3, PARKIN), and the degradation of the autophagosome cargo (CTSL1). They also showed that autophagy was directly correlated to the severity of muscle atrophy by evidencing an inverse correlation with the thigh cross-sectional area [182]. Finally, another study reported an increase in the phosphoULK1/ULK1 ratio—ULK1 is a kinase protein essential for autophagy initiation and progression [183].

Autophagy is tightly connected to other catabolic pathways in skeletal muscle, such as the ubiquitin–proteasome system under the common control of FOXO3. Indeed, it has been shown that FOXO3 overexpression in C2C12 myotubes, as well as in isolated mouse muscle fibers, enhanced proteolysis mainly via lysosomal degradation [186]. Moreover, it has also been reported that increased autophagy was directly linked to oxidative stress, as treatment with an antioxidant molecule decreased autophagy levels in cultured myotubes [184].

#### 9.1.5. Other Catabolic Pathways

Two other pathways allow a protein degradation in skeletal muscle, namely Ca^2+^-dependent (role of calpain) and caspase-dependent (leading to apoptosis), usually taking the first steps of protein degradation by a disruption of the myofibrillar assembly. Few data are available regarding their implication in muscle wasting in COPD specifically. To our knowledge, only one study has investigated the calpain expression in COPD quadriceps and reported an unchanged expression compared to that of the control group [187]. Apoptosis has also been discussed above (cf. mitochondrial dysfunction).

### 9.2. Protein Synthesis

In skeletal muscle, protein synthesis is mainly mediated by the IGF/Akt/mTOR pathway, which seems globally decreased, although contradictory data between mRNA and protein levels of different components of the cascade have been reported.

For example, a study performed on the quadriceps of 91 COPD patients showed that Akt phosphorylation was decreased in COPD compared to healthy subjects as well as in sarcopenic versus nonsarcopenic patients. However, other markers of protein synthesis signaling such as S6- or 4EBP1-phosphorylations were increased in both cases [188]. Of note, this study did not evidence any increased activity of the ubiquitin–proteasome pathway in COPD or sarcopenic patients. Later, the same team found nevertheless an increase in Akt signaling in COPD quadriceps [183]. In the quadriceps of exacerbating COPD patients, IGF-1 was found downregulated at the mRNA level, as well as myoD at the protein level [28].

Meanwhile, some studies report a counterintuitive rise of IGF-1 or Akt levels [155,165], possibly due to a compensatory mechanism.

**Figure 2:**
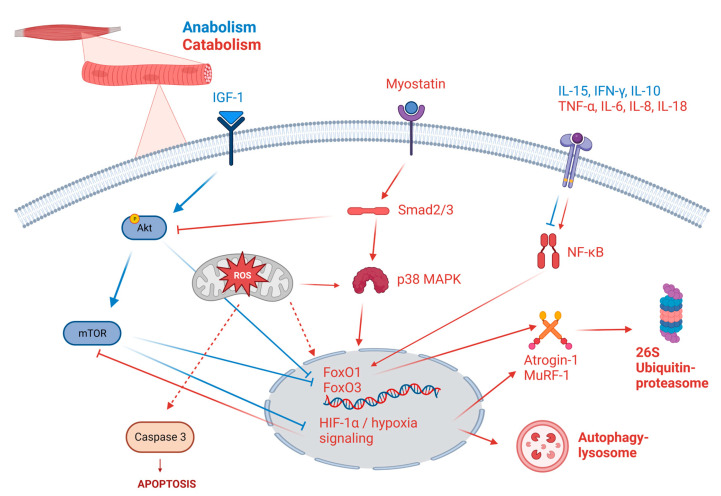
Alteration of the hypertrophy/atrophy balance leading to impaired protein turnover in COPD myocytes. Anabolic (hypertrophic) processes are represented in blue and catabolic (atrophic) in red. The chosen pathways have been identified as dysregulated in COPD patients or experimental models of COPD. IFN, interferon. IGF, insulin growth factor. IL, interleukin. MAPK, mitogen-activated protein kinase. Created with BioRender.com (accessed on 1 February 2023).

Altogether, the balance between protein synthesis and degradation is altered in sarcopenic COPD patients in a complex way (Figure 2). A recent systematic review emphasizes the lack of consistency between reported data on gene expression in skeletal muscle [189]. Discrepancies between studies might come from the complex interconnection between pathways, as well as the patients’ selection and the fact that biopsies are taken at different time courses of the disease. Indeed, muscle mass regulation is highly dynamic and timelapse studies of in vivo models might help to decipher the underlying mechanisms. However, experimental models do not reflect the complexity of human disease, as currently illustrated in the field of cancer research [190], and are quite heterogeneous in their ability to reproduce every characteristic of COPD sarcopenia. Indeed, skeletal muscle tissue alterations in rodent models are inconsistent, partly due to the diversity of the experimental procedures used (CS exposure, elastase or lipopolysaccharide instillations, transgenic models, etc.). Moreover, one could argue that rodent models are not perfectly adapted to study skeletal muscle dysfunction, given the difference between rodent and human muscle tissue—for example, four different types of myofibers exist in mice (type I, IIa, IIx, IIb) whereas only three in humans (lacking type IIb). Table 1 gathers and summarizes the existing models as well as the functional, histological, and molecular alterations reported.

Finally, it seems that less attention has been paid to protein synthesis pathways in COPD muscle, although they represent an interesting therapeutic pool, as strategies aiming to block protein degradation have not been proven effective yet [177].

## 10. Conclusions

Skeletal muscle wasting is a crucial issue for COPD patients, both associated with an impaired quality of life and a significant mortality. The underlying pathophysiology is vastly multifactorial and implies both extrinsic and intrinsic factors. A complex interplay between mitochondrial dysfunction and metabolic alteration leads to an impaired muscle mass regulation. It is likely that the different pathogenic processes are intertwined, and all converge to an increased catabolism.

One of the major difficulties lies in the interindividual heterogeneity between subtypes of COPD patients (e.g., cachectic emphysematous patients as opposed to obese patients with muscle atrophy). Establishing profiles in skeletal muscle dysfunction is thus essential to tailor COPD treatment in a personalized manner. Cues might come from a vast transcriptional analysis in the muscle identifying distinct molecular signatures among COPD patients, associated to specific profiles, for example in response to pulmonary rehabilitation [222].

Better defining the time course of muscle wasting is also a crucial issue to understand the molecular basis and draw new potential therapeutic leads. In this regard, experimental models can help study every stage of muscle wasting, provided that their functional and histological characteristics fit those of the disease.

COPD must be considered as a systemic disease, and deciphering mechanisms leading to muscle wasting should help to treat the disease in a personalized manner. Effective physical therapy should be tailored to each patient. The intensity should be sufficient to induce anabolism but not too intense in order to limit oxidative stress damage. On a short-term perspective, clinical research should focus on finding clinical markers determining the individual part of each discussed risk factor in sarcopenia, in order to prevent skeletal muscle wasting intensification. In a longer-term perspective, translational research should aim at precising the dysregulation of the different anabolic and catabolic pathways according to the disease evolution and patient profile. New therapeutic targets could also emerge thanks to the use of large-scale multiomic approaches, such as spatial transcriptomics [223], allowing the spatiotemporal identification of new molecular targets in skeletal muscle health and disease.

## Figures and Tables

**Table 1:** Characterization of locomotor skeletal muscle dysfunction in COPD rodent models.

Animal	Model/Toxic Agent	Modalities (Exposure, Administration)	Skeletal Muscle Function Alterations	Histological Alterations	Molecular Alterations	Ref.
Mouse	Exposure to CS	Whole body (durations: from 4 weeks to 6 months)	Decreased muscle mass and force (grip strength, endurance, isolated contractility)	Decreased capillarity and myofibers CSA, fiber-type switch (type I => II)	Increased catabolism (whole tissue mRNA), protein carbonylation Decreased citrate synthase activity	[32,41,141,191,192,193,194,195,196,197]
		Nose-only (from 8 weeks to 6 months)	Decreased muscle mass; decreased strength	Fiber-type switch		[198,199]
		IP injection		Decreased myofibers CSA		[200]
	Elastase	Intratracheal (1X to 5X)	Decreased muscle mass and endurance	Decreased myofibers’ CSA (type I), fiber-type switch		[201,202]
	Elastase + LPS	x3 each	Decreased muscle mass and grip strength		Slightly increased catabolism (whole tissue protein)	[203]
	LPS	Intratracheal	Decreased muscle mass		Increased catabolism (mRNA and protein level)	[204,205]
	Bleomycin	Intratracheal		Decreased myofibers’ CSA	Increased catabolism (protein level)	[206]
	Transgenic mouse	TNF-α+/+	Decreased muscle mass and exercise capacity, increased ex vivo fatigue, decreased in vivo regenerative capacity	Decreased myofibers’ CSA	Increased catabolism (mRNA level), decreased citrate synthase activity	[42,207]
		IL-13+/+	Decreased muscle mass, grip strength and contractility	Decreased myofibers’ CSA	Increased catabolism (mRNA level)	[208]
		Klotho−/−	Decreased grip strength and endurance			[209]
Rat	CS	Whole body (duration: from 4 weeks to 4 months ½)	Decreased grip strength and contractility	Decreased myofibers’ CSA	Increased catabolism (mRNA level) Decreased mitochondrial density and abnormal morphology	[210,211,212,213]
	CS + elastase	CS: whole body 13 weeks Elastase: intratracheal	Decreased mass	Decreased myofibers’ CSA, fiber-type switch	Increased catabolism (protein level)	[214]
	CS + Klebsiella pneumonia	CS: whole body 20 weeks Klebsiella pneumonia instillations, every 5 days for the first 8 weeks			Increased tissular apoptosis and decreased oxidative phosphorylation	[215]
	CS + LPS			Decreased myofibers’ CSA	Decreased mitochondrial density, altered ultrastructure and oxidative capacity	[216,217]
	IL-6 administration	7 days subcutaneous	Decreased muscle mass	Decreased myofibers’ CSA		[218]
Guinea Pig	CS	Acute exposure (24 h)			Decreased skeletal muscle glutathioneIncreased plasma lipid peroxidation	[219]
Hamster	Elastase		Decreased muscle mass	Decreased myofibers’ CSA		[220,221]

CSA: cross-sectional area; CS: cigarette smoke; IP: intraperitoneal.

## Data Availability

Not applicable.
